# Supplementary material for: Neuropsychology and Electroencephalography in Rural Children at Neurodevelopmental Risk: A Scoping Review
Source: Pediatr Rep. 2023 Dec 12;15(4):722–40. doi: 10.3390/pediatric15040065 (PMC10747224; doi:10.3390/pediatric15040065)
Supplement: Supplementary file 1 [file pediatrrep-15-00065-s001.zip › pediatrrep-2621311-supplementary.pdf]

Manuscript title

Neuropsychology and Electroencephalography in Rural Children at Neurodevelopmental Risk: A Scoping Review

Section

2. Methodology
2.1. Search strategy

Manual search keywords used:

("rural" AND "eeg" AND "children")
("rural" AND "risk factors" AND "children")
(“neurodevelopment” AND “risk factors”)
(“neurodevelopment" AND “malnutrition” AND “EEG”)
